# Resveratrol reduced the detrimental effects of malondialdehyde on human endothelial cells

**DOI:** 10.34172/jcvtr.2021.27

**Published:** 2021-04-24

**Authors:** Mehdi Hassanpour, Çıgır Biray Avci, Reza Rahbarghazi, Aysa Rezabakhsh, Alireza Nourazarian, Elahe Nabat, Farzaneh Fathi, Majid Khaksar

**Affiliations:** ^1^Stem Cell Research Center, Tabriz University of Medical Sciences, Tabriz, Iran; ^2^Department of Biochemistry and Clinical Laboratories, Faculty of Medicine, Tabriz University of Medical Sciences, Tabriz, Iran; ^3^Department of Medical Biology, Faculty of Medicine, Ege University, Izmir, Turkey; ^4^Department of Applied Cell Sciences, Faculty of Advanced Medical Sciences, Tabriz University of Medical Sciences, Tabriz, Iran; ^5^Cardiovascular Research Center, Tabriz University of Medical Science, Tabriz, Iran; ^6^Pharmaceutical Sciences Research Center, Ardabil University of Medical Sciences, Ardabil, Iran

**Keywords:** Human Endothelial Cells, Resveratrol, Malondialdehyde, Chromatin Remodeling, Diabetes Mellitus

## Abstract

***Introduction:*** According to the statistics, vascular injury occurs during the onset of diabetic changes after the production of several byproducts. Many authorities have focused to find an alternative therapy for diabetic patients. In this study, we investigated the therapeutic effects of natural polyphenol like resveratrol on human endothelial cells exposed to malondialdehyde for 48 hours.

***Methods:*** Human Umbilical Vein Endothelial Cells were randomly classified into four groups;control, malondialdehyde (2.5 mM), resveratrol (100 μM), and cells received the combined regime for 48 hours. Cell viability was determined by 3-(4, 5-dimethyl thiazol-2-yl) 2, 5-diphenyl-tetrazoliumbromide (MTT) assay. Griess reaction was performed to measure the content of Nitric oxide (NO).Apoptosis was studied by using real-time polymerase chain reaction (RT-PCR) and western blotting assays. Levels of receptor tyrosine kinases like VEGFR-1, -2, Tie-1, and -2 were analyzed by enzyme-linked immunosorbent assay(ELISA). The affinity of resveratrol and malondialdehyde to serum albumin was measured by Surface Plasmon Resonance Assay. Any changes in chromatin remodeling were detected by PCR array analysis.

***Results:*** Resveratrol reduced cytotoxicity and NO content inside cells induced by malondialdehyde(MDA) (*P* < 0.05). Endothelial cell apoptosis was decreased by the reduction of pro-apoptotic factor Bax and increase of Bcl-2 following the incubation with resveratrol (*P* < 0.05). MDA-induced receptor tyrosine kinases increase was inhibited by resveratrol and reached near-to-normal levels (*P* < 0.05).Surface Plasmon Resonance revealed a higher affinity of resveratrol to albumin compared to the malondialdehyde-albumin complex. Polymerase chain reaction (PCR) array revealed the potency of resveratrol in chromatin remodeling following the treatment with malondialdehyde (*P* < 0.05).

***Conclusion:*** Based on our findings, resveratrol has the potential to decrease diabetic vascular injury induced by lipid byproducts such as MDA.

## Introduction

In light of changes in lifestyle patterns, the prevalence of type 2 diabetes mellitus (DM) is increasingly growing up globally.^1^ Uncontrolled modification in the bioactivity of endothelial cells (ECs) seen during the diabetic changes contributes to micro- and macro-vascular complications.^2,3^ The increase of systemic glucose levels along with insulin resistance can contribute to impaired vasodilation/vasoconstriction, arterial stiffness, enhanced cytokine expression, and activation of coagulation factors.^4^ Additionally, tissue accumulation of byproducts such advanced glycation end products and malondialdehyde (MDA) by lipid peroxidation are responsible for the loss of normal function in the cardiovascular system.^5,6^ For instance, some data have shown that MDA exhibited mutagenic effects on the genome.^7^ Due to prominent electrophilicity, MDA can directly attack proteins and DNA, leading to cellular damages.^7^ It has been proposed MDA could react with nucleosides and formed adducts to deoxyguanosine and deoxyadenosine which alter genome sequence.^8^ Prolonged exposure of ECs to MDA increases the possibility of atherosclerosis and pro-thrombotic cascade via the activation of nicotinamide adenine dinucleotide phosphate (NADPH) oxidase.^9^

In addition to hormone therapy, regular exercise program, and amendment of food regime, many attempts have been collected to find a therapeutic approach with a low rate of side effects and high-throughput therapy rate. Phytomedicine is touted as an alternative method for curing various diseases, used from ancient times to the present.^10^ The compound 3, 5, 4′-trihydroxystilbene, known also as (Resveratrol) Res, with a polyphenol structure is extensively found in many plants and fruits.^11^ A large number of biological activities, such as anti-cancer, antioxidant, neuroprotective, hepatoprotective, antiviral, anti-inflammatory, anti-hypertensive activities has been documented concerning the application of Res in *in vitro* and *in vivo* conditions.^12^

Res was found to initiate an anti-oxidant activity in human keratinocytes by engaging the AMP-activated kinase/Forkhead box O3 pathway, contributing cell resistance against senile changes.^13^ In line with this claim, Res potentially preserves and regulates the oxidative status in human cardiomyocytes undergone oxidative stress.^14^ Considering the existence of various experiments related to the anti-diabetic activity of Res,^15^ different beneficial outcomes, and signaling pathways should be defined. In the context of diabetes, it was found that Res could increase the intracellular cargo of glucose, amplify insulin activity via the Akt/PKB pathway on the target cell type.^16^ Under the diabetic condition, the valuable effect of Res on different tissues correlates with the up-regulation of AMPK and SIRT1.^17^ In ECs, Res could decrease the detrimental effect of the diabetic condition by preserving mitochondrial activity via the modulation of Sirt3 signaling, an activator of sirtuin 1 and an increased activity of oxidative stress enzymes.^18^ The pro-inflammatory response of Res was found to decrease by the suppression of interleukin-8 and down-regulation of surface adhesion molecules ICAM and VCAM-1 meanwhile the level of C-reactive protein and tumor necrosis factor-α decreased in condition with Res.^19^

In the current experiment, the blunting effect of Res was investigated in human ECs exposed to MDA. We also wanted to show that whether Res treatment could decrease different cytotoxic effects induced by MDA on endothelial lineage in the level of chromatin.

## Material and Method

### Cell culture

Human Umbilical Vein Endothelial Cells (HUVECs) (NCBI code: C554) were purchased from the National Cell Bank of Iran (Pasteur, Iran). We used Dulbecco′s Modified Eagle′s Medium/high glucose (DMEM/HG) (Gibco) supplemented with 10% fetal bovine serum and 1% Penicillin-Streptomycin (Biosera). Cells were kept in a humidified atmosphere with 5% CO_2_ at 37°C. Trypsin-EDTA solution (Dilution: 0.25%, Gibco) was used to subculture the cells. Cells at passage 3 to 6 were used for subsequent analyses.

### Dose determination and in vitro cell survival assay

To examine the different concentrations of Res, ranging from 25, 50, 75, 100, 125, 250, 500, 750, and 1000 µM, on HUVECs, we performed a conventional MTT assay. Briefly, HUVEC (1 × 10^4^ cells per well of 96-well-plates) were cultured for 7 days. Following the experimental period, cells were treated with a different dosage of Res for 48 h. Thereafter, the supernatant medium was discarded and 50 µL of MTT solution (Dilution: 5 mg/mL; Sigma) was added to each well. After 4 h incubation time, 200 µL dimethyl sulfoxide (Merck) solution was added and the absorbance was determined at 490 nm by using a microplate reader (Bio-Tek). The data were obtained from the three sets of experiments each done in octuplicate. In the next stage, we selected a non-toxic dose of Res (100 µM) for subsequent analyses. In the current experiment, we classified the cells into four groups; Group I: control; Group II: cells received 2.5 mM MDA; Group III: cell treated with 100 µM Res and Group IV: Cells given the combination of MDA and Res. The cells were treated for 48 h and the therapeutic effect of Res on MDA-induced cell cytotoxicity determined by MTT assay.

### Measuring NO content

The production of NO was determined by using Griess reagent according to previous protocols ^20^. Shortly, NO is converted into a more stable metabolite nitrite by Griess reaction which is further transformed to nitrous acid. The combination of nitrous acid with sulfanilamide ultimately forms an Azo dye. For this purpose, 1 × 10^4^ cells were plated per well of 96-well-plates and incubated with Res, MDA, or their combinations for 48 h. The NO content of each group was determined by Griess reagent at 540 nm and expressed as nM/mL.

### Quantitative Real-Time PCR

We also determined the effect of MDA, Res, and their combination on the expression of Bax and Bcl-2. 48 hours after treatment, the total RNA content was isolated by using 1 mL RNX-plus buffer (Cat No: MR7713C; CinnaGen) according to the manufacturer’s recommendation. Subsequently, 200 μL chloroform was loaded and samples were centrifuged at 12000 rpm for 15 minutesmin at 5°C. Then, the upper phase that contains RNA and DNA was mixed with an equivalent volume of isopropanol and incubated at 4°C for 15 minutes. After centrifugation, the supernatant was discarded, the pellet was dissolved in 1 mL of 75% ethanol solution and the harvested RNA dissolved in 50 µL DEPC water. The total content of RNA from each sample was determined by using a NanoDrop (Thermo Scientific). Samples were treated with DNase1 kit (Cat no: en0521; Fermentaz) to exclude possible genomic DNA contamination. Before real-time PCR analysis, total RNA was transcribed reversely by the Bioneer kit (Cat no: K-2046) and cDNA was synthesized. We used Oligo 7 software for primer designing (Table 1). The qRT-PCR reaction was performed with the cDNA, SYBR premix ExTaq kit (Cat no: RR820L; TaKaRa), and target gene primers, and a Rotor-Gene Corbett System. Data were analyzed by the Pfaffl method and normalization was done with the housekeeping gene glyceraldehyde 3-phosphate dehydrogenase (GAPDH). The experiment was performed in triplicate.

**Table 1:** Primer list

**Gene**	**Sequence**	**Tm (°C)**
*Bax*	F: 5’-TGCCAGCAAACTGGTGCTCA-3’ R: 5’-GCACTCCCGCCACAAAGATG-3’	59
*Bcl-2*	F: 5’- CCTGTCGATGACTGAGTACC-3’ R: 5’-GAGACAGCCAGGAGAAATCA-3’	55
*GAPDH*	F: 5’-TTGACCTCAACTACATGGTTTACA-3’ R: 5’-GCTCCTGGAAGATGGTGATG-3’	59

### Western blotting

Western blot analysis was done to evaluate apoptosis status by monitoring protein levels of Bax and Bcl-2 in HUVECs from different groups. For protein lysis, cells were collected and lysed in ice-cold cell lysis buffer solution contained protease inhibitor cocktails (Sigma) and centrifuged at 14000 rpm for 20 minutes at 4°C. Total protein content was determined in the supernatant by using the PicoDrop spectrophotometer (Model: PICOPET01). To resolve protein samples, 100 μg of cell lysate was loaded in the lanes of 12% SDS-polyacrylamide gel and then transferred to PVDF membrane (Millipore). The membranes were incubated with specific antibodies against Bcl-2 and Bax (Dilution: 1:1000, both from Abcam) at 4°C overnight. The next day, blots were incubated with the HRP-conjugated anti-rabbit IgG antibody for 1 h (Abcam). The immune-reactive bands were determined by using the ECL solution (Roche). Using the HP ScanJet G31110 system, we scanned the x-ray films. Densitometry evaluation of bands was done with ImageJ software ver.1.44p. We used β-actin as an internal loading control.

### Detection of RTKs such as VEGFR-1, -2, Tie-1 and Tie-2 levels by ELISA

To assess the effect of MDA plus Res on RTKs (receptor tyrosine kinases) content, we measured the levels of VEGFR-1, -2, Tie-1, and Tie-2 using ELISA. These receptors are expressed on the ECs and control angiogenesis signaling cascades^21^. RTKs levels were detected using the ELISA method designed by our team. A panel of antibodies against Tie-1 (Cat no: ab27851, Abcam), Tie-2 (Cat no: ab24859, Abcam), VEGFR-1 (Cat no: ab32152, Abcam), and VEGFR-2 (Cat no: ab39256, Abcam) were used. Polystyrene 96-well plates (SPL) were filled with 100 µL (dilution: 1 µg/mL) of each antibody per well and kept overnight at 4°C. The next day, 1% bovine serum albumin (BSA) was added and plates were maintained for 1 h at RT and then incubated with 100 µL of diluted samples containing an equal amount of protein. Following twice washing with PBS (each for 5 minutes), 100 µL of anti-mouse (dilution 1: 2000) and -rabbit HRP conjugated secondary antibodies (dilution 1: 4000) was added (both purchased from Abcam). A chromogenic solution containing 3% tetramethylbenzidine (Sigma) was used for visualization and the reaction stopped by adding 50 µL of 5% H_2_SO_4_. The absorbance was recorded at 450 nm. The content of each factor was calculated based on a standard curve generated from samples of known peptides.

### Investigating the interaction of Res and MDA by SPR method

To measure the possible physical interaction of Res with MDA, we used the surface plasmon resonance (SPR) technique. SPR method is a very specialized optical system to display changes in the refractive index and mass in the vicinity of a sensor surface at the aqueous layer. A multi-parameter SPR device (MP-SPR Navi 210A, BioNavis Ltd, Tampere-region, Finland) uses the Kretschman prism configuration having two flow channels were applied to the investigation of kinetic parameters. In line with this statement, the BSA molecule was immobilized on a gold sensor surface and the second component including MDA and Resveratrol (RES) in five different concentrations at µM ranges injected on the BSA surface. Carboxymethyl Dextran chips with carboxy dextran layer on the gold surface were used for BSA attachment by 1-ethyl-3-(3-dimethylaminopropyl) carbodiimide/N-hydroxysuccinimide (EDC/NHS) coupling method. A flow rate of 30 μl/min was used throughout the experiments with a sensor temperature fixed at 27°C in running buffer PBS at pH 7.4. After activation of carboxylic groups on Carboxymethyl Dextran chip via EDC/NHS, BSA (0.25 mg/mL) was attached to the surface through covalent amide binding formation^22^. For blocking free sites of Carboxymethyl Dextran surface 1 M ethanolamine was used. The immobilization level and response unit (RU) of BSA was 0.15 RU. Trace DrawerTM for SPR NaviTM was applied for calculations of affinity and kinetics of the measured interaction.

### PCR Array

The possible effect of MDA on HUVECs chromatin remodeling was investigated by a PCR array. RNAs were extracted from each group and reverse-transcripted to cDNA (Qiagen). The Human Epigenetic Chromatin Remodeling Factors PCR Array (cat no: PAHS-086Z) Signaling RT^2^ Profiler^TM^ PCR Array was performed to monitor the expression of 84 key genes involved in chromatin remodeling under experimental condition (Supplementary Table S1). PCR arrays were performed on the Light Cycler 480 Instrument II (Roche). The housekeeping genes ACTB, B2M, GAPDH, HPRT1, and RPLP0 were used to normalize the amount of RNA. Fold change values were calculated using the formula: 2-^ΔΔCt^. RT2 Profiler PCR Array results were analyzed with RT2 Profiler PCR Array Version 3.5 data analysis software (SABiosciences, USA). Differences more than ± 2 fold change expression accepted as the cut-off value. *P* < 0.05 were considered statistically significant.

### Statistical analysis

The data were represented as mean ± SD in this study. To find the statistical analysis between groups, we used One-Way ANOVA and Tukey post-hoc analysis by using GraphPad InStat^TM^ software version 2.02. *P* < 0.05 was considered significant.

## Result

### The effects of Res and MDA in HUVECs viability

**Figure 1:**
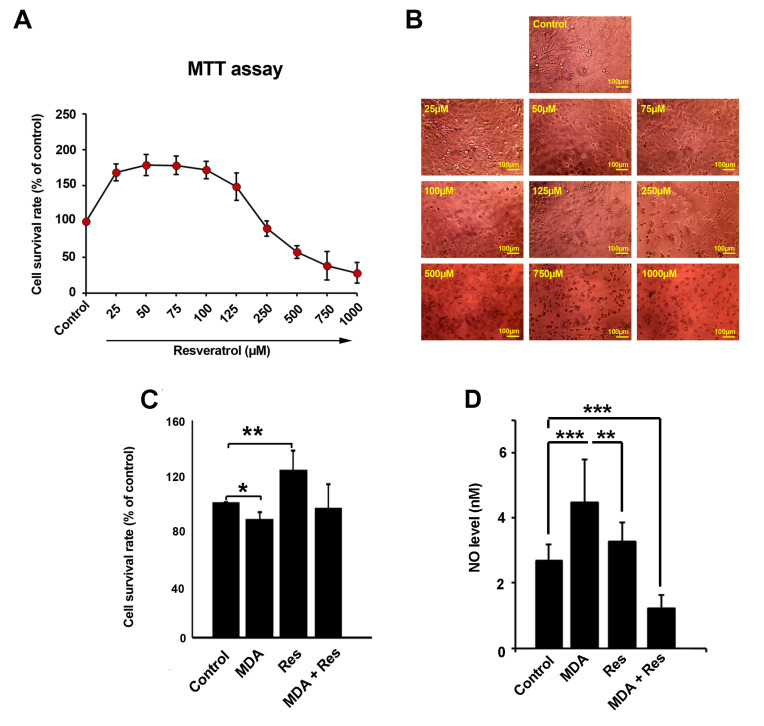
The dose-dependent activity of Res was shown by MTT assay (**A**). In doses below 250 µM, Res increased the HUVECs proliferation and viability. Microscopic bright field examination of HUVECs after treatment with various doses of Res (**B**). Res had the potential to inverse the cytotoxic effect of MDA on HUVECs done by MTT (**C**). Measuring NO level in HUVECs from group MDA, Res, and MDA + Res (**D**). Griess reaction indicates an increase in NO production following treatment with 2.5 mM MDA while Res closes this value to near normal content. One-Way ANOVA and Tukey post-hoc (n=3). *P < 0.05; **P < 0.01; and ***P < 0.001

Corroborating to our MTT results, Res could change the survival rate of HUVECs in a dose-dependent manner (Figure 1A). According to our data, the exposure of HUVECs to different doses of Res below 200 µM (ranging from 25 to 125 µM) increased the survival rate (Figure 1A). The most stimulatory effects of Res on HUVECs were recorded for cells from groups 50 and 75 µM (Figure 1A). Notably, HUVECs treatment with 250 µM Res or higher doses resulted in cell cytotoxicity. The high rate of cell death was correlated with 1000 µM Res (Figure 1A). Microscopic examination revealed the detachment of HUVECs exposed to higher doses of Res while the concentrations below 100 µM Res yielded in condensed cell layer (Figure 1B). These results highlight the fact that Res could modulate HUVECs’ survival rate dose-dependently. For subsequent analyses, we treated HUVECs with 100 µM Res. According to the MTT assay, the treatment of HUVECs with 2.5 mM MDA reduced cell viability compared to the control (*p*_Control VS. MDA_ <0.05; Figure 1C). The simultaneous treatment of HUVECs with the combined regime of MDA and Res, improved cell survival rate, and closed it near-to-normal level (*P* > 0.05). No statistically significant differences were found between the MDA and MDA + Res groups (*P* > 0.05). These data demonstrated that Res possibly could, in part, blunt the toxicity of MDA on endothelial lineage.

### An increased NO level was decreased in MDA-treated ECs after treatment with Res

Based on the results from Griess analysis, we found an increase in the intracellular level of NO in ECs after treatment with 2.5 mM MDA (*p*_Control VS. MDA_ < 0.001; Figure 1D). Although the level of NO was increased in Res treated cells, but non-significant differences were obtained compared to the control (*P* > 0.05; Figure 1D). ECs treatment with Res had the potential to reduce nitrosative stress exposed to MDA by decreasing the intracellular content of NO and even reached the levels below the control (*P* < 0.001). According to these data, one could hypothesize that Res could reduce the detrimental effect of MDA in diabetic conditions on ECs by modulating the production of NO.

### Res reversed the apoptotic effect of MDA on HUVECs

**Figure 2:**
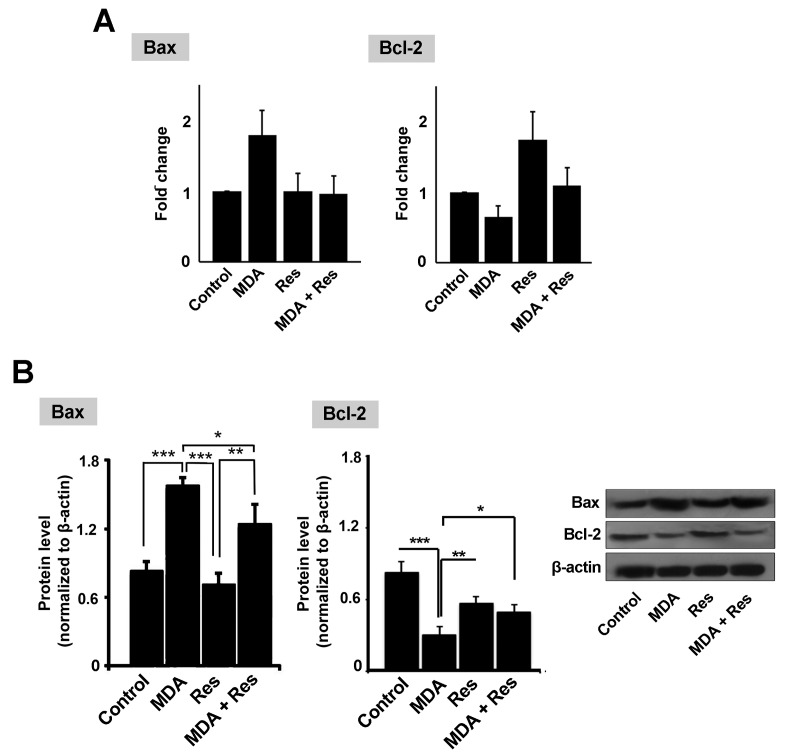
Real-time PCR analysis of pro-apoptotic and anti-apoptotic genes in cells from MDA, Res, and MDA + Res (**A**). Western blotting assay (**B**). Data indicate that MDA promotes Bax expression while decreases Bcl-2 levels, showing the increase of cells entering apoptosis. Res decreased the apoptosis by blunting the MDA effect on Bcl-2 and Bax. One-Way ANOVA and Tukey post-hoc (n=3). *P < 0.05; **P < 0.01; and ***P < 0.001

Real-time PCR analysis revealed that MDA increased the transcription level of the pro-apoptotic gene Bax (~ 1.8-fold) compared to the control HUVECs (Figure 2A). No differences were found regarding the mRNA level of the Bax gene between Res and the control. The addition of Res to MDA-treated HUVECs blunted the apoptotic feature of MDA and reached Bax level to near normal content (Figure 2A). Compared to Bax, we found that Res initiated opposite effects on the expression of the anti-apoptotic gene Bcl-2 (Figure 2A). According to our data, Res alone or in combination with MDA increased the mRNA level of Bcl-2 compared to MDA-treated HUVECs. These data highlight the anti-apoptotic effect of Res on endothelial lineage after the onset of diabetic changes.

### Res inhibited the factors mediating apoptosis

Western blotting demonstrated that the level of Bax (a pro-apoptotic factor) was increased following the treatment with MDA (*p*_Control versus MDA_< 0.001) while the exposure of these cells with Res could decrease the protein level of Bax (*p*_Res versus MDA_< 0.05; Figure 2B). Similar to the Bax level, we also determined the protein level of Bcl-2 by using Western blotting (Figure 2B). The results confirmed the reduced Bcl-2 level induced by MDA was increased following the treatment with Res (*p*_Res versus MDA_< 0.01; Figure 2B). These data showed that Res has the potency to reduce the apoptosis rate in conditions with high MDA content by modulating the expression of Bcl-2 and Bax.

### Res decreased the increased level of RTKs induced by MDA

**Figure 3:**
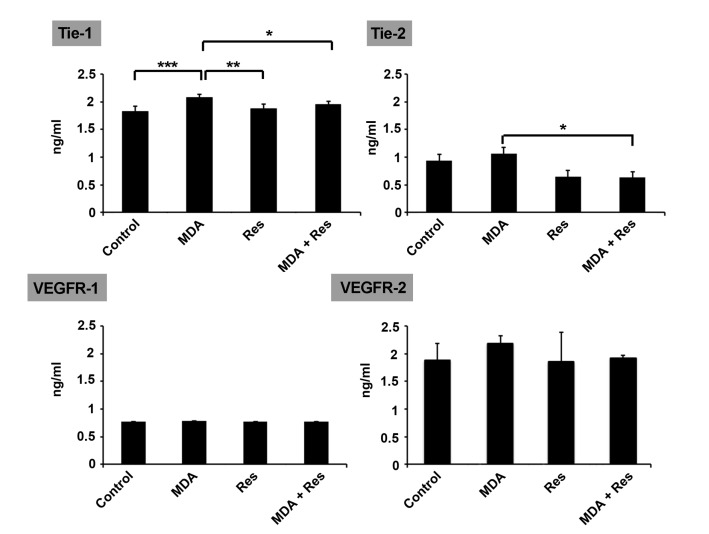
Measuring the level of RTKs by ELISA assay. ELISA indicates that MDA increases Tie-1, -2, and VEGFR-2 but no significant effects on VEGFR-1. The treatment of HUVECs with Res returned the values to near control levels. One-Way ANOVA and Tukey post-hoc (n=12). *P < 0.05; **P < 0.01; and ***P < 0.001

According to data from ELISA, we found that an increased level of RTKs in HUVECs under treatment with MDA (Figure 3). In a better word, levels of Tie-1, Tie-2, and VEGFR-2 receptors were increased after exposure to MDA (Tie-1: *p*_Control versus MDA_< 0.001). We found that the increased levels of RTKs by 2.5 mM MDA were decreased following treatment with 100 µM Res and reached near-normal levels (*P* < 0.05; Figure 3). No significant differences were observed related to the level of VEGFR-1 and -2 in HUVECs from different groups (*P* > 0.05). These data support the notion that the exposure of HUVECs with MDA, a diabetes byproduct, could increase RTK levels.

### SPR revealed the interaction of Res with MDA

**Figure 4:**
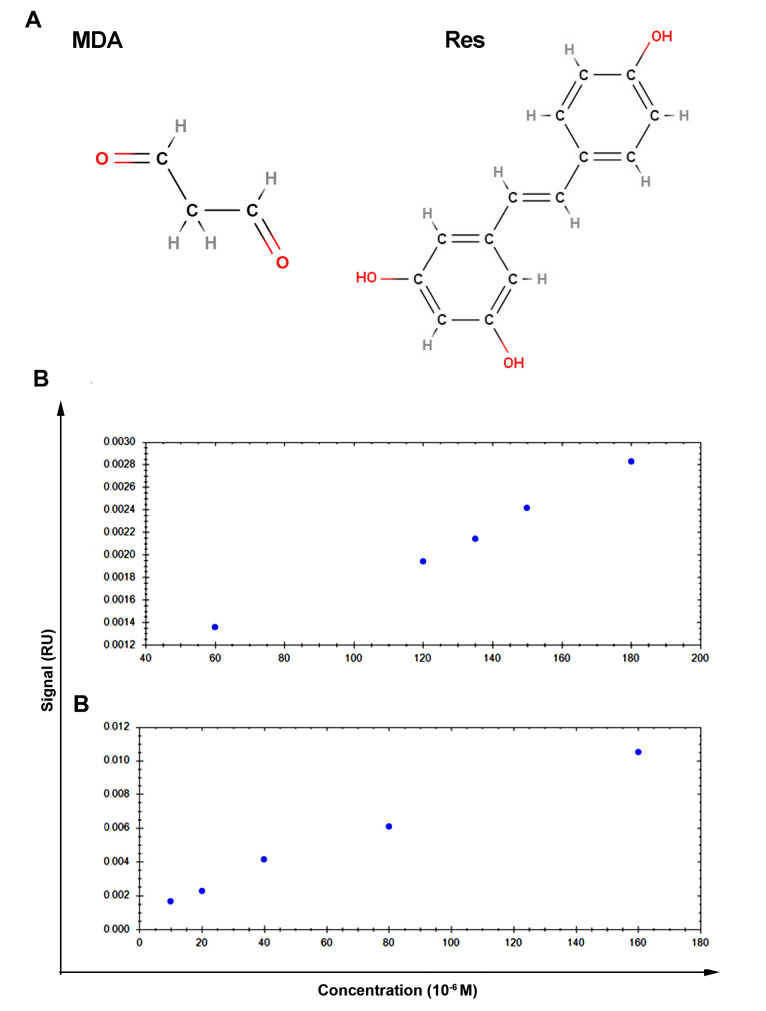
The molecular structure of MDA and Res. SPR analysis of MDA and Res interaction with BSA (**A-B**). The curve of affinity MDA (**A:** KD: 3.05 ×10^-4^ M) and Res (**B:** KD: 2.25× 10^-4^) with BSA (n=3).

SPR method was used to measure the interaction of Res and MDA with the serum albumin (BSA).^23,24^ Trace DrawerTM for SPR NaviTM was applied for calculations of affinity and kinetics of the measured interaction. At pH 7.4 the affinity unit (K_D_) for RES and MDA were 2.25 × 10^-4^ M and 3.05 × 10^-4^ M, respectively (Figure 4A). The affinity of Res-BSA with a low K_D_ is more than MDA with a high K_D_. Because of having more functional groups in Res like OH groups that are shown in Figure 4B, this molecule can form strong interaction with BSA that refers to hydrogen bonding - a relatively strong form of intermolecular attraction- in comparison to MDA. In general, high-affinity analyte binding causes a stronger intermolecular force between the analyte molecule and BSA while low-affinity involves less intermolecular force between the analyte and its receptor. Commensurate with these data, one could hypothesize that Res possibly has more half time in blood compared to the MDA, because of Res-BSA interaction.

### PCR array

To evaluate the possible modulatory effect of Res and/or MDA on epigenetic chromatin remodeling, we performed PCR array analysis regarding the relevant signaling pathway. According to data from PCR array, MDA increased the expression of different genes from Helicase/DNA-Binding Domain (CHD), Polycomb Group, and Homeodomain signaling transduction such as *CDYL* (7.64-fold), *CHD2* (7.82-fold), *CHD7* (8.37-fold), *PHF1* (8.87-fold), and *TRIM27* (3.21-fold) while the mRNA content of other Helicase and Bromodomain genes such as *CDYL2* (-6.63-fold), *BPTF* (-2.24-fold) was decreased compared to the non-treated HUVECs (*P* < 0.05; Table 2 and Supplementary Figure 1). In HUVECs treated with the combined regime of MDA and Res, up-regulation of *CDYL* (7.51-fold), *CHD2*(7.16-fold), *CHD7* (7.44-fold), and *PHF1* (8.39-fold) was found that coincided with the reduction in the transcription level of genes *PHF7* (-7.86-fold), *TRIM27*(-5.23-fold), *BRWD1* (-2.12-fold; a Bromodomain factor), and *SMARCA2* (-7.02-fold; an SWI/SNF Complex Component) (*P* < 0.05; Table 2 and Supplementary Figure 1). In cells treated with Res alone, we detected an elevated transcription in genes such as *CDYL* (6.8-fold), *CHD2* (6.16-fold), and *PHF1* (7.69-fold). Res had potential to down-regulate the expression of *BRD7* (-7.33-fold; a Bromodomain factor), *CBX7* (-7.59-fold; a factor from Heterochromatin Protein 1 (HP1) Homologs), *CHD9* (-9.29-fold), *INO80* (-8.24-fold), *PCGF2* (-9.2), *PHF5A* (-3.74-fold), *BAZ2A* (-2.21-fold), *BRD1* (-7.73-fold), *CBX3* (-7.93-fold), *CHD5* (-4.41-fold), ING2 (-7.97-fold), NAB2 (-9.2-fold), PHF2 (-6.98-fold), SUZ12 (-8.59-fold), BRD2 (-2.16-fold), ING3 (-2.55-fold), BRD3 (-2.32-fold), CBX5 (-7.62-fold), and WDR11 (-8.83-fold) (*P* < 0.05; Table 2 and Supplementary Figure 1). These results demonstrate that both MDA and Res can change the expression of genes involved in chromatin remodeling.

**Table 2:** Gene expression profiling of chromatin remodeling signaling in HUVECs following treatment with Res and MDA.

**Gene**	**Res**	**MDA**	**MDA + Res**	**Gene**	**Res**	**MDA**	**MDA + Res**	**Gene**	**Res**	**MDA**	**MDA + Res**
*ARID1A*	-1.67	-0.57	-0.80	*RNF2*	-0.87	-0.31	-0.85	*PHF21A*	-1.63	0.53	-0.81
*BRD7*	-7.33	0.25	-1.03	*BAZ2B*	-1.51	-0.69	-1.82	*TRIM27*	0.77	3.21	-5.23
*CBX7*	-7.59	1.46	-0.59	*BRWD1*	-1.75	-1.04	-2.12	*BRD3*	-2.32	1.34	-0.38
*CHD9*	-9.29	0.65	-0.98	*CHD2*	6.16	7.82	7.16	*CBX5*	-7.62	0.02	-0.04
*INO80*	-8.24	0.79	-0.97	*EZH2*	-1.21	-0.17	-0.76	*CHD7*	-1.02	8.37	7.44
*PCGF2*	-9.20	0.70	-1.07	*MECP2*	-1.19	0.30	-0.74	*ING4*	-1.72	-0.17	-0.65
*PHF5A*	-3.74	0.46	-0.54	*PHC2*	-0.94	0.03	-0.99	*PBRM1*	-1.22	-0.08	-1.25
*ASXL1*	-1.10	-0.85	-1.19	*SMARCA2*	-1.99	-0.72	-7.02	*PHF21B*	-1.00	-0.72	-0.51
*BRD8*	-1.25	-0.70	-0.98	*BMI1*	-0.92	-0.01	-1.23	*WDR11*	-8.83	0.68	-0.64
*CBX8*	-1.61	-0.55	-1.14	*BRWD3*	-0.70	-1.25	0.53	*BRD4*	1.54	0.47	1.10
*CTBP1*	-0.04	0.44	-0.51	*CHD3*	-1.00	0.39	-0.41	*CBX6*	-0.57	0.62	-0.65
*MBD1*	-0.57	-0.31	-0.87	*HINFP*	-1.30	0.33	-0.75	*CHD8*	-1.56	-0.04	-1.22
*PCGF3*	0.02	-1.38	0.17	*MTA1*	-1.17	0.94	-0.52	*ING5*	-0.99	-0.04	-0.67
*PHF6*	-0.62	-0.07	-0.55	*PHF1*	7.69	8.78	8.39	*PCGF1*	-1.25	0.68	-0.51
*BAZ1A*	-1.32	-0.25	-1.53	*SMARCA4*	-1.99	-0.04	-1.12	*PHF3*	-0.29	0.72	-0.21
*BRDT*	-1.66	-1.08	-1.54	*BPTF*	-1.48	-2.24	-1.71	*ZMYND8*	-1.00	0.58	-0.57
*CDYL*	6.80	7.64	7.51	*CBX1*	-0.17	-0.08	0.15				
*CTBP2*	-0.85	0.47	-0.31	*CHD4*	-1.63	-0.22	-1.16				
*MBD2*	-0.71	0.11	-0.69	*ING1*	-1.34	0.10	-1.06				
*PCGF5*	-0.72	-0.26	-0.54	*MTA2*	-1.13	0.41	-0.44				
*PHF7*	-0.59	0.11	-7.86	*PHF13*	-1.63	-0.03	-0.80				
*BAZ1B*	-0.99	0.37	-1.07	*SPEN*	-0.72	0.56	-0.43				
*BRPF1*	0.72	0.39	0.45	*BRD1*	-7.73	-0.74	-0.68				
*CDYL2*	-1.22	-6.63	-1.53	*CBX3*	-7.93	0.07	-0.46				
*CTCF*	-1.34	-0.11	-0.96	*CHD5*	-4.41	1.54	1.05				
*MBD3*	-0.98	0.18	-1.00	*ING2*	-7.97	-0.70	-0.75				
*PCGF6*	-1.49	-0.82	-1.13	*NAB2*	-9.20	-0.62	-0.61				
*RING1*	0.17	-1.33	-0.71	*PHF2*	-6.98	0.26	-1.08				
*BAZ2A*	-2.21	-0.16	-1.10	*SUZ12*	-8.59	-1.33	-0.32				
*BRPF3*	-0.57	0.17	-0.10	*BRD2*	-2.16	0.39	0.01				
*CHD1*	-1.75	0.70	-0.61	*CBX4*	-0.38	1.23	0.50				
*EED*	-1.14	0.19	-1.17	*CHD6*	-1.91	0.33	-0.95				
*MBD4*	-1.30	-0.07	-1.15	*ING3*	-2.55	-0.05	-1.13				
*PHC1*	-1.61	-1.14	-1.41	*NSD1*	-1.42	0.36	-0.77				

The *P* values are calculated based on a Student’s t-test of the replicate 2^(-DeltaCt)^ values for each gene in the control group and treatment groups, and *P* values less than 0.05 are indicated in red for genes with 2 fold increase and blue for genes with -2 fold decrease (n=3).

## Discussion

To date, many attempts have been collected to alleviate the detrimental effects of diabetic conditions and byproducts on the cardiovascular system. In the current experiment, we aimed to detect the therapeutic effects of Res on MDA-induced angiopathy in the endothelial lineage. Regarding the Res concentration used in the current experiment, both inhibitory and stimulatory effects of Res was recorded. Consistent with our data, the dose-dependent action of Res has been elucidated previously.^25^ In lower doses Res alone acts as a cytoprotective agent while the use of higher doses suppresses cell function and activity.^25^ To investigate the therapeutic effects of Res on condition with high-content MDA, we subjected HUVECs to non-toxic doses (100 µM). Based on our results, Res had the potential to attenuate the detrimental effects of MDA and reached the cell survival rate to near-normal levels. Chen and colleagues previously demonstrated that treatment of HUVECs with 10 µM Res decreased the pro-inflammatory status of these cells by increasing cell resistance by the promotion of autophagy effectors MAP1LC3B2 and SQSTM1/p62.^26^ Additionally, it was proved that Res *per se* reduced diabetes-related cytopathies via the modulation of intracellular glucose transport and activated insulin signaling through the Akt/PKB signaling pathway.^16^ Data showed a reduction in NO level of MDA-treated HUVECs post-treatment with 100 µM Res, showing the decrease of nitrosative stress under diabetic conditions.^27,28^ One explanation would be that the activation of and PI3-K)/Akt (MAPK)/ERK signaling pathways are involved in the induction of endothelial nitric oxide synthase.^28^ We also found the beneficial cytoprotective effect of Res on HUVECs was related to the inhibition of pro-apoptotic factor Bax and the promotion of anti-apoptotic agent Bcl-2.^25,29^ Despite the anti-apoptotic activity of Res on HUVECs exposed to the high dose of MDA, previous authorities declared that higher doses of Res could initiate apoptotic changes in cancer cells via the induction of chromatin changes, RNA, and DNA synthesis inhibition, and cycle arrest in cells located at G1 and S phases, confirming the dual opposite effects of Res on distinct cell type.^25^ The expression of RTKs such as Tie-1, -2, VEGFR-1, and -2 seems to accelerate the angiogenic response in ECs in different conditions however abnormal decrease/increase of these receptors could also abrogate the normal function of neovascularization.^30-32^ Besides, the alteration of multiple neovascularization stimulating factors such as VEGFR and other RTKs could lead to excessive retinal vascular leakage and other vascular disorders.^33^ Based on our results, Res could decrease the increased level of RTKs induced by MDA and closed them to near normal contents. Some experiments showed the regulatory potency of Res on pathological angiogenesis following the onset of various diseases by the modulation of eukaryotic elongation factor-2 kinase.^34^ Also, the promotion of adhesion molecules such as connexin-43 by Res acts as a prohibitory stimulus to control the dysregulated proliferation of ECs.^35^ The treatment of stem cells with Res caused stabilized capillary-like structures coincided with an enhanced expression of VE-cadherin and regulation of MiR-21/Akt/β-Catenin.^36^ One interesting result that originated from this study was the determination of MDA and/or Res association with BSA. According to data, we found an increased affinity of Res to BSA compared to the MDA-BSA complex. The higher affinity of Res to serum albumin showed an active bio-distribution of Res to different sites of the body meanwhile prohibited the long-term viability of MDA inside the body. It was previously found that MDA and byproducts from lipid peroxidation could enthusiastically attach to a cell membrane protein. It seems that the higher Res affinity to serum proteins could attenuate the toxic effects of MDA.^37^

PCR array analysis showed that MDA has the potential to alter the expression of multiple genes from different signal transduction pathways involved in chromatin remodeling. Based on our finding the expression of CDYL, CHD2, CHD7, PHF1, TRIM27 was up-regulated in HUVECs after 48 hours. Among these genes, CDYL, CHD2, CHD7 belong to Chromodomain/Helicase/DNA-Binding Domain (CHD) Proteins transduction pathway and exhibit ATP-dependent activity and could regulate nucleosome assembly. The alteration in the expression of these genes allows the access of the transcriptional machinery system to DNA.^38^ The up-regulation of PHF1, a member of Plant Homeodomain (PHD) Proteins signal transduction pathway, in MDA-treated HUVECs shows the alteration of chromatin structure and changes in the expression of diverse genes.^39^ TRIM27 is located in the nuclear matrix and suppress gene expression with the collaboration of polycomb proteins.^40^ These data showed that MDA could disrupt the integrity of nucleosomes thus changing the spatial organization of heterochromatin and euchromatin. Along with these changes, the transcription ability seems to be altered as compared to the non-treated control cells. Overexpression of *PHF1* in MDA-treated groups may correlate with the activation of P53 and apoptotic changes.^41^ We found that treatment of HUVECs with the combination of Res and MDA returned *TRIM27*to the minimum level comparable to the control group.^42^ Based on data, Res down-regulated the expression of numerous genes related to chromatin integrity during the onset of diabetes-related cytopathies. For instance, the total transcription level of genes *BRD7*, *CBX7*, *CHD9*, *INO80*, *PCGF2, CHD7, PHF5A,* and *BAZ2A* was suppressed in HUVECs in the Res-treated cells. Along with these changes, the expression of BPTF was significantly inhibited in MDA-treated cells compared to the control group. This gene belongs to Bromodomain Proteins signal transduction pathway which participates in the regulation of transcription.^43^ Bromodomain proteins are involved in a diverse range of functions, such as acetylating histones, remodeling chromatin, and recruiting other factors necessary for transcription.^44^ According to our data, we found that the expression of above-mentioned genes was near-to-control levels in groups received Res plus MDA. These data confirmed the chromatin remodeling and acetylation of amino acid residue in histones governed by Res. The expression of genes like *WDR11, CDYL,*and *CHD2*in MDA-treated HUVECs showed promoted acetylation rate in the genes. Meanwhile over activation of these genes stimulate different signaling pathways correlated with apoptosis and cell cycle.^45^ Whether MDA acts differently in multiple genes belonging to each signal transduction pathway is the subject of debate. There are number of nuclear and cytoplasmic factor involved in the regulation of cell growth.^46^ Here, we found that MDA inhibited the expression of both *ING2* and *3,*inhibitor of growth (ING) family members, after 48 hours. The increase of ING2 and 3 expressions in Res + MDA showed the removal of the MDA inhibitory effect on the growth of family membrane genes by Res. The exact relevance of MDA to the chromatin remodeling and function of genes in the genome remains elusive. Here, we showed that MDA could alter the expression of different genes related to multiple signal transduction pathways. Res blunted the MDA effect on the chromatin remodeling and close the changes to near-normal levels.

## Conclusion

Overall, this study highlighted the therapeutic effect of Res on diabetes-related microvascular pathologies induced by MDA. The more beneficial effects of Res on diabetic micro- and macro-vascular pathologies must address by the conduction of further experiments in vivo and in vitro milieu.

## Acknowledgments

We kindly appreciate the personnel of the Stem Cell Research Center for guidance and help.

## Competing interests

Authors declare no conflict of interest related to this study.

## Ethical approval

All phases of this study were approved by a Local Ethics Committee of Tabriz University of Medical Sciences (IR.TBZMED.VCR.REC.1399.196).

## Funding

This study was supported by a grant from Tabriz University of Medical Sciences.

## Supplementary materials

Supplementary file contains Supplementary Figure S1 and Supplementary Table S1.
