# Supplementary material for: Resveratrol reduced the detrimental effects of malondialdehyde on human endothelial cells
Source: J Cardiovasc Thorac Res. 2021 Apr 24;13(2):131–40. doi: 10.34172/jcvtr.2021.27 (PMC8302894; doi:10.34172/jcvtr.2021.27)
Supplement: Supplementary file 1 — Supplementary file contains Supplementary Figure S1 and Supplementary Table S1. [file jcvtr-13-131-s001.pdf]

Supplementary file

Table S1. The expression of different genes from multiple signal transduction pathways

Gene List of different genes from multiple Signal transduction pathways

SWI / SNF Complex Components<br><br>**ARID1A, INO80 (INOC1), PBRM1, SMARCA2, SMARCA4.**
Polycomb Group Genes<br>**ASXL1, BMI1 (PCGF4), CTBP1, CTBP2, EED, EZH2, PCGF1, PCGF2 (RNF110), PCGF3, PCGF5, PCGF6, PHC1, PHC2, RING1, RNF2, SUZ12, TRIM27.**
Chromobox / Heterochromatin Protein 1 (HP1) Homologs<br>**CBX1, CBX3, CBX4, CBX5, CBX6, CBX7, CBX8.**
Bromodomain Proteins<br>**BAZ1A, BAZ1B, BAZ2A, BAZ2B, BPTF, BRD1, BRD2, BRD3, BRD4, BRD7, BRD8, BRDT, BRPF1, BRPF3, BRWD1, WDR11 (BRWD2), BRWD3, ZMYND8.**
Chromodomain / Helicase / DNA-Binding Domain (CHD) Proteins<br>**CDYL, CDYL2, CHD1, CHD2, CHD3, CHD4, CHD5, CHD6, CHD7, CHD8, CHD9.**
Nucleosome-Remodeling & Histone Deacetylase (NuRD) Complex Components<br>**CHD3, MBD3, MTA1, MTA2, NAB2, SPEN.**
Plant Homeodomain (PHD) Proteins<br>**NSD1, PHF1, PHF2, PHF3, PHF5A, PHF6, PHF7, PHF13, PHF21A, PHF21B.**
Inhibitor of Growth (ING) Family Members<br>**ING1, ING2, ING3, ING4, ING5, RING1.**
Methyl-CpG DNA Binding Domain (MDB) Proteins<br>**MBD1, MBD2, MBD3, MBD4, MECP2, HINFP.**
CCCTC-Binding Factor (Zinc Finger Protein)<br>**CTCF.**

was investigated using PCR array analysis.

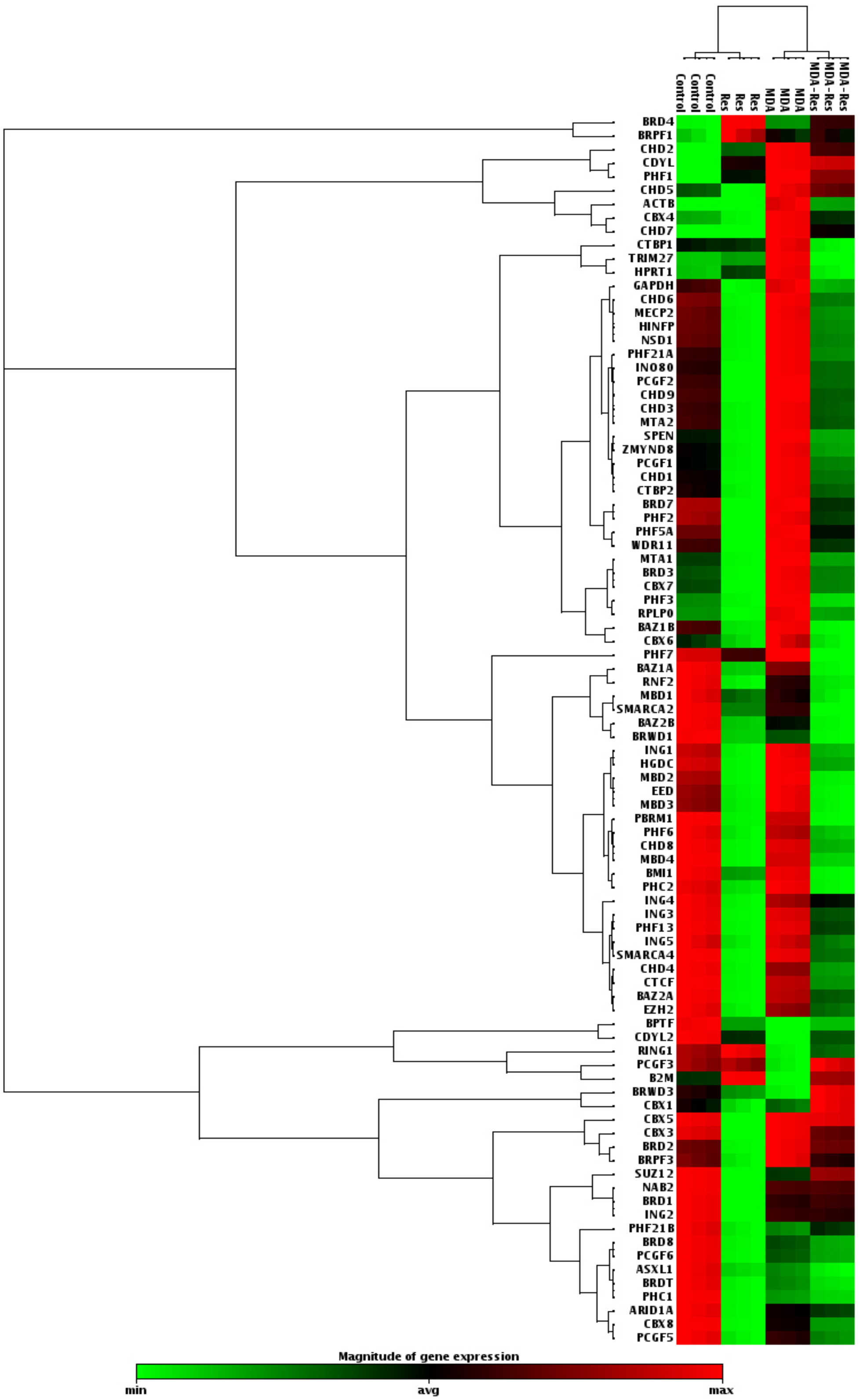

Figure S1. Clustogram representative of PCR array analysis of chromatin remodeling (n=3)
